# Transient autoantibodies to danger signals

**DOI:** 10.3389/fimmu.2023.1046300

**Published:** 2023-01-18

**Authors:** Elana R. Shaw, Polly Matzinger

**Affiliations:** ^1^ Medical Scientist Training Program, School of Medicine, Johns Hopkins University, Baltimore, MD, United States; ^2^ Ghost Lab, Division of Intramural Research, National Institute of Allergy and Infectious Diseases, National Institutes of Health, Bethesda, MD, United States

**Keywords:** type 1 interferon, autoantibodies, danger model, danger signal, autoimmunity, virus

## Abstract

The Danger Model predicts that there are some molecules that no immune system can ever be fully tolerant of, namely proteins that are only transiently expressed during times of stress, infection, or injury. Among these are the danger/alarm signals themselves. Accordingly, a fleeting autoantibody response to danger signals is expected during times when they are released. Depending on context, these autoantibodies may serve beneficial “housekeeping” functions by removing surplus danger signals from the circulation or, conversely, create an immunodeficiency. Here, we will focus on the Type 1 Interferons as examples of foreseeable targets for a transient autoantibody response, but the principles outlined should hold for other danger-associated molecules as well.

## 1 Introduction

**Figure 1:**
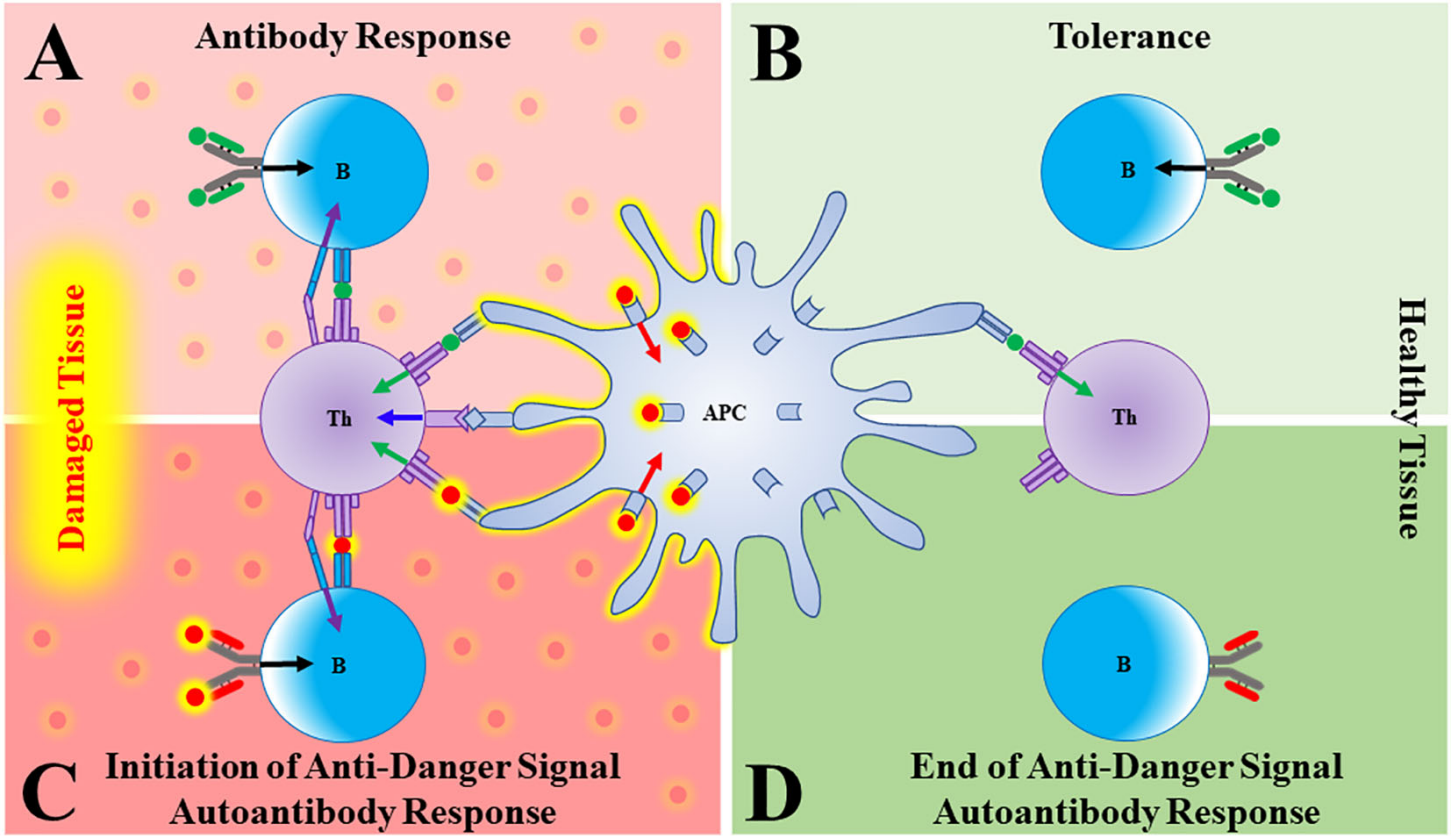
Autoantibodies to danger signals. **(A)** In damaged/distressed tissue, an antigen presenting cell (APC) that has been activated by a danger signal (red sphere, red arrow) presents antigen (green sphere) to a T cell, providing T cell Signal 1 (green arrow) and co-stimulation (blue arrow). The T cell can now give a “help” signal (purple arrow) to a B cell that had bound its cognate antigen (green sphere, black arrow), leading to a productive antibody response. **(B)** In the absence of danger signals (such as in healthy tissue), APCs are not activated and both T and B cells that bind antigen go on to die, thus generating tolerance. **(C)** As danger signals are elaborated in response to damage, a B cell bearing a B cell receptor (BCR) that binds the danger signal itself will become activated and produce antibody. **(D)** As the tissue recovers, danger signals wane. A B cell specific for danger signals will therefore go unstimulated and the anti-danger signal autoantibody response will fade.

Danger signals (such as DNA, RNA, ATP, heat shock proteins, hyaluron breakdown products, inflammatory cytokines, HMGB1, interferons, etc.) are molecules that are fleetingly released as a result of infection, injury, toxins or other forms of non-physiological cell stress or death (1). They also represent a unique set of self-proteins that are predicted to be targets for an autoimmune response. To explain why, we start with two primary assumptions of the Danger Model (2, 3), namely that,

1. Antigen presenting cells (APCs) are primarily activated to induce immune responses by danger signals that have been released from stressed, damaged, or infected tissues (**Figure 1A**).
2. Tolerance is induced to any molecule (foreign or self) that is present and persists in the absence of activated APCs (**Figure 1B**).

Most forms of clinically overt autoimmunity are uncommon because activation of self-directed immune responses requires simultaneous failures of central and peripheral tolerance. While the thymus promotes central tolerance to self-proteins through clonal deletion of immature autoreactive T cells, the thymus cannot induce tolerance to antigens that are not found there (4–6). Thus, peripheral tolerance mechanisms exist to eliminate autoreactive B and T cells that bind peripheral self-antigens in the absence of danger-activated APCs (**Figure 1B**).

We propose that an autoimmune response to danger signals is expected because 1) it should be difficult to induce central tolerance to self-molecules that are produced transiently and 2) peripheral tolerance mechanisms will necessarily fail to generate tolerance towards danger signals because danger signals themselves can activate APCs.

## 2 Prediction

Let’s use the Type 1 interferons (T1IFNs) as an example. These molecules are produced only transiently (often in response to infection), and so are not normally present. Therefore, just like lymphocytes specific for pathogens, newly produced T1IFN-reactive T or B cells in a healthy individual would circulate as unactivated naïve cells due to lack of Signal 1. We would predict, however, that during an episode where T1IFNs are released/elaborated in response to danger, these naïve cells would encounter both T1IFN (Signal 1) and activated APCs (Signal 2), resulting in the production of anti-T1IFN autoantibodies **(** **Figure 1C** **)**. Once the damage instigating agent has been cleared, T1IFNs stop being produced, activated APCs die, and the autoimmune response should wane as T1IFN-reactive T and B cells die or revert to a memory state, ready to respond to the next insult **(** **Figure 1D** **).**

## 3 Evidence

When central tolerance is disturbed, as it is in patients with thymic malignancies or with genetic or acquired deficiencies in autoimmune regulator (AIRE), neutralizing anti-T1IFN autoantibodies are common (7–11). Similar IgG autoantibodies that neutralize T1IFNs can also result from infections. For example, they were recently identified in 10-20% of patients with critical COVID-19 (12). Studying the temporal dynamics of anti-T1IFN autoantibodies, we found that autoantibody levels remained stable in patients with AIRE deficiency and thymic malignancies, but were highly dynamic in patients with acute COVID-19 (13). In the COVID-19 patients, both the levels and the neutralizing activity of autoantibodies peaked shortly after disease onset and then rapidly declined to undetectable levels during convalescence. Our data suggested that the increase in T1IFN production during the early stages of viral infection triggered a short-lived response of high titer neutralizing autoantibodies in these patients.

## 4 Perhaps a purpose

We started to wonder: If complete central and peripheral tolerance to transiently expressed danger signals would be nearly impossible using the logic of the Danger Model, could there be a function for these autoreactive cells and antibodies? Decades ago, Mel Cohn suggested that germline-encoded autoreactive “housekeeping antibodies” served the important role of clearing autogenously generated waste products from the extracellular space (14). One could envisage that the continued presence of danger signals long after an infection is cleared could lead to prolonged and unnecessary immune responses – both wasteful and potentially dangerous. Thus, transient autoantibodies directed against danger signals could be an evolutionarily selected characteristic. Indeed, binding (non-neutralizing) anti-IFN-γ autoantibodies that wax and wane in response to viral disease have been observed since 1990 (15) and transient anti-HMGB1 autoantibodies emerge in some patients with septic shock and are highly predictive of survival (16). In patients with systemic lupus erythematosus, neutralizing anti-IFN-α autoantibodies were found to have positive effects akin to those of therapeutic anti-IFN monoclonals (17). Similarly, endogenous disease-suppressing autoantibodies that neutralize TNF-α have been found in some patients with rheumatoid arthritis (18). These findings support the idea that the immune system has evolved to generate autoantibodies for the purpose of restraining immunopathology.

## 5 Thoughts: Anti-T1IFN autoantibodies in viral disease

It has become popular to consider that neutralizing anti-T1IFN autoantibodies underlie susceptibility to severe viral diseases (11, 12, 19–22). However, recent data with COVID-19 pokes a hole in this view. Specifically, Meisel and colleagues identified several patients with AIRE deficiency who experienced mild COVID-19 disease despite having high levels of potently neutralizing anti-T1IFN autoantibodies (23). An alternative view could be that these autoantibodies instead may be helpful disease modifiers, for example, by neutralizing or encouraging phagocytosis of excess cytokine to prevent hyperinflammation during the later stages of COVID-19. Supporting this idea is a study on COVID-19 patients admitted to the ICU where Abers et al. found that 10-week survival was higher in patients with autoantibodies (5 out of 26 = 19.2%) than in patients without (11 out of 192 = 5.7%) (24). Thus, “housekeeping” anti-cytokine autoAbs may play an important and underexplored role in the host response to viral disease. In other cases, they may simply be epiphenomena caused by a perfect storm of high levels of T1IFNs, activated APCs and rare(ish)? autoreactive lymphocytes.

## 6 Conclusions

The Danger Model predicts that transient cytokines that can activate APCs are particularly logical targets for an autoantibody response, and that a short-lived autoantibody response to danger signals might occur during any infection/injury in otherwise healthy individuals. Depending on context and neutralization activity, anti-danger signal autoantibodies may benefit the host by restraining immunopathology or, conversely, provoke an immunodeficiency (25, 26) or autoimmune disease. Further study of anti-danger signal autoantibodies may provide novel insights into how the immune system regulates itself and how derangements in this process may lead to disease.

## Data availability statement

The original contributions presented in the study are included in the article/Supplementary Material. Further inquiries can be directed to the corresponding author.

## Author contributions

Writing – original draft, ES. Writing – review, suggestions, and editing, ES and PM. All authors contributed to the article and approved the submitted version.
